# A compact and power efficient full adder-subtractor layout in QCA technology

**DOI:** 10.1371/journal.pone.0335789

**Published:** 2025-11-10

**Authors:** Ali H. Majeed

**Affiliations:** Department of Electrical Engineering, Faculty of Engineering, University of Kufa, Najaf, Iraq; Mustansiriyah University, IRAQ

## Abstract

The pursuit of miniaturizing digital circuits and reducing power consumption has focused attention on non-traditional computing technologies. Among these technologies, quantum dot cellular automata (QCA) stand out as a promising alternative to conventional CMOS chips, offering high-density designs with minimal power dissipation. This paper presents a novel QCA layout that unifies full addition and subtraction operations within a single, compact structure. Simulation outcomes, derived through QCADesigner software, affirm the proposed circuit’s operational integrity, stable behaviour, and design efficiency. The proposed architecture demonstrates significant improvements, offering 6.7%, 25%, and 30% reductions in cell count, area, and cost, respectively, compared to the best-reported design. Furthermore, the total energy savings achieved by the proposed design are approximately 6%, 4%, and 6% at tunnel energies of 0.5 EK, 1 EK, and 1.5 EK, respectively, compared to its counterparts. This approach not only demonstrates functional versatility but exhibits high integration potential for larger quantum cellular automata-based computational units, representing a step forward in the development of efficient nanoscale computing architectures.

## Introduction

As the semiconductor industry approaches the limits of CMOS technology, it becomes increasingly difficult to overcome challenges such as power dissipation, device density, and propagation time. [1]. As the growth in processor capabilities slows down in accordance with Moore’s Law, researchers have begun exploring alternative technologies that could lead to a new generation of computing systems. Among these technologies, quantum dot cellular automata (QCA) stands out as one of the most promising approaches [2]. It introduces a radically different computational mechanism that relies on the position of the electron within the quantum dots rather than the flow of current.

**Fig 1:**
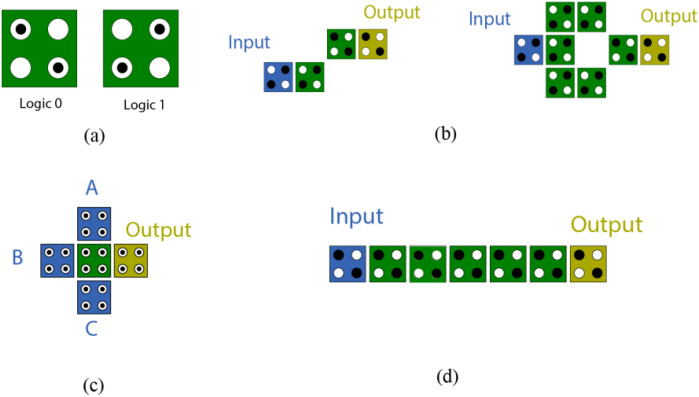
The basic blocks in QCA Technology (a) cell layouts (b) inverter configurations (c) majority voter and (d) QCA wire.

QCA technology relies on three basic components: The Inverter, the majority voter, and the QCA wire. These are the fundamental components upon which all logic operations are built. The inverter flips the polarity of the inputs; the majority gate performs the decision-making between three inputs, forming the basis for AND, OR, and more complex functions; and the wire enables data transfer between components by propagating the polarization across a linear series of QCA cells [3]. Fig 1 illustrates the basic technology components, which represent the building blocks upon which all high-level QCA circuits are built. A deep understanding of how these modules interact is important for designing optimized, low-area, and energy-efficient structures in nanoscale circuits.

QCA enables the design of nanoscale logic circuits with high speeds up to Terahertz [4]. The circuit structure is of great importance, as its counterparts are compared based on the area occupied by the structure, the number of cells, latency and the cost (which mainly depends on the above metrics). Therefore, researchers aim to design circuits in various structures and configurations to achieve the optimal metrics [5]. Among these circuits, the full-adder plays a pivotal role in arithmetic logic units (ALUs), data paths, and processors, making its efficient design critical for QCA-based systems.

In parallel, QCA subtractor designs have received less attention. Many subtractors are realized through circuit extensions of adders. However, these often lead to an increase in gate count and spatial complexity. More recently, dual-function arithmetic circuits capable of performing both addition and subtraction have been explored, but such designs tend to involve additional logic layers or suffer from performance bottlenecks due to increased wire length and cell usage. Given the critical importance of layout compactness in QCA, particularly in power and area-constrained environments, there remains a clear need for functionally versatile yet spatially efficient arithmetic designs. This work addresses this gap by proposing a novel QCA-based full adder/subtractor circuit that combines both operations within a single, compact layout. The design minimizes the number of cells, avoids wire crossovers entirely, and operates under standard four-phase clocking. Through simulation and comparison with state-of-the-art designs, the proposed circuit offers significant improvements, offering several advantages in terms of occupied area, cell count, and cost, making it a strong candidate for integration into future nanosystems.

Despite efforts to design compact QCA computational circuits, most designs either neglect their functionality or involve complex, multi-layered, and rotated cell structures, which pose manufacturing challenges. This gap will be addressed in this work by presenting a simplified, multifunctional design suitable for practical nanoscale integration.

The remainder of this paper is organized as follows: Section 2 provides the reader with sufficient information on the history of the full adder in QCA. Section 3 introduces the proposed full adder/subtractor framework. Section 4 provides simulation results and comparative evaluation. Finally, Section 5 presents the conclusions drawn from this study.

## Related works

In the digital world, the full adder circuit is the cornerstone of computation, often forming the foundation for more complex processing units such as arithmetic and logic units (ALUs) and multipliers. Their structural efficiency and operational accuracy directly impact the speed and efficiency of digital systems. Therefore, optimizing full adder designs in quantum dot cellular automata (QCA) has remained a topic of great interest in academic and industrial research. Many researchers have investigated methods to improve full adder circuits by reducing design complexity, particularly the number of QCA cells used, as well as minimizing the chip area occupied and reducing the response time caused by signal propagation delays.

### Full adder designs in QCA

Over the years, several QCA-based full adder designs have been proposed to enhance logic density, reduce area, and minimize delay in arithmetic operations.

The first landmark attempt to implement a full adder in QCA was made by Tougaw et al. in 1994 [6]. Their design featured a single-layer structure consisting of 192 cells, relying heavily on five main logic gates and three inverters. While this pioneering structure demonstrated the feasibility of arithmetic logic in quantum computing, it was significantly large and did not optimize in area or speed by contemporary standards. Nevertheless, it provided an important foundation upon which future work would be built.

A major shift toward simplification occurred years later when Zhang et al. developed a new layout of FA using only three majority gates [7]. This architecture provided a simple and intuitive design methodology that reduced the number of gates, thereby paving the way for smaller, more efficient designs. It also highlighted the potential of simpler methods to preserve functionality while improving manufacturability.

In 2007, Azghadi et al. introduced a new perspective by employing a five-input majority gate as the central logic unit in their full adder proposal [8]. This modification allowed for more logic to be encapsulated within a single gate, thereby reducing circuit depth and enhancing processing speed. Building on this concept, Navi et al. (2010) implemented a practical QCA layout using a multilayer approach [9]. Their work demonstrated how the added design complexity of multilayer fabrication could be leveraged to reduce wire congestion and enhance signal routing in densely packed layouts.

**Fig 2:**
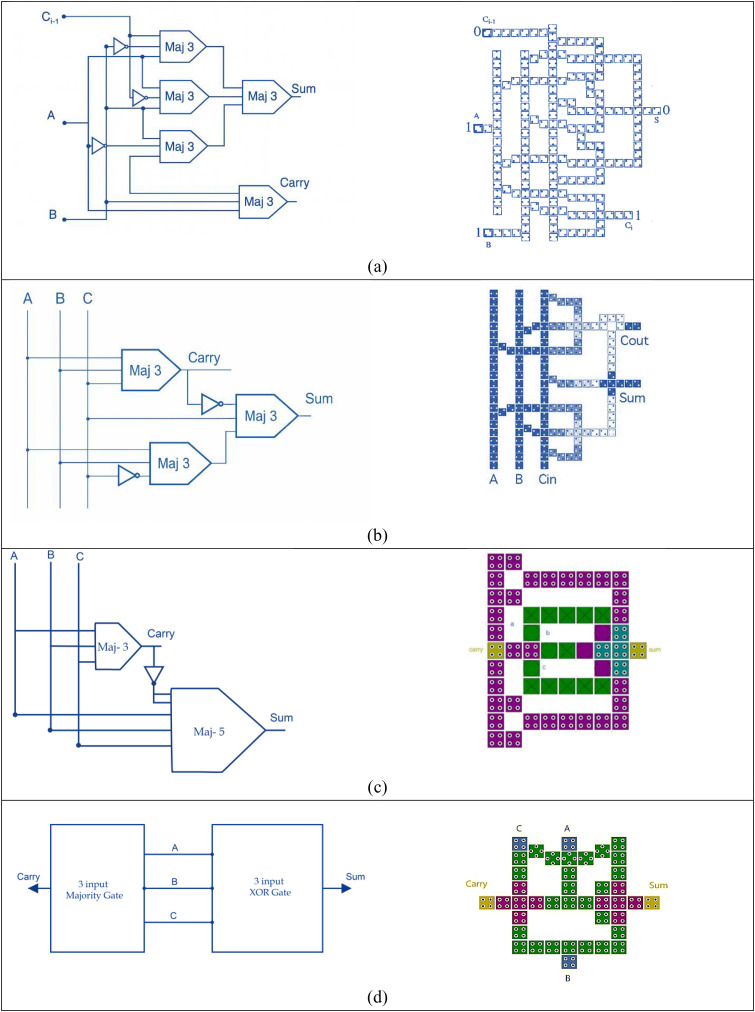
The block diagram and QCA layout of FA introduced in (a) [6], (b) [7], (c) [9] and (d) [10]).

Another pivotal advancement emerged in 2016 when Ahmad et al. proposed a highly innovative design for a three-input XOR gate that departed from traditional Boolean logic formulations [10]. Instead of expressing the XOR operation through standard logic synthesis, they capitalized on the inherent electrostatic interactions among QCA cells to achieve the desired behavior. This strategy allowed for a far more compact and structurally elegant full adder layout. Their work showcased the potential of QCA as a technology not only for replicating conventional logic but also for reimagining how logic operations could be physically realized in nanoscale environments. Fig 2 shows the block diagram and QCA layout of the above-mentioned adder circuits.

### Subtractor designs in QCA

While significant efforts have been directed toward improving full adder circuits in QCA, research into subtractor architectures, particularly those optimized for compactness and speed, has remained relatively limited. Among the notable contributions, Pudi and Sridharan [11] proposed a new design for a single-bit subtractor utilizing double inverters and three majority gates, achieving an impressive latency of 0.75 clock cycles, where each cycle required 4 clock phases (switch, hold, release and relax). Following this, many papers were introduced using the 5-bit majority gate. Resht and Banday [12] introduced a more complex multilayer QCA-based full subtractor consisting of 104 cells, occupying an area of 0.1043 µm² and exhibiting a latency of 1.75 clock cycles.

**Fig 3:**
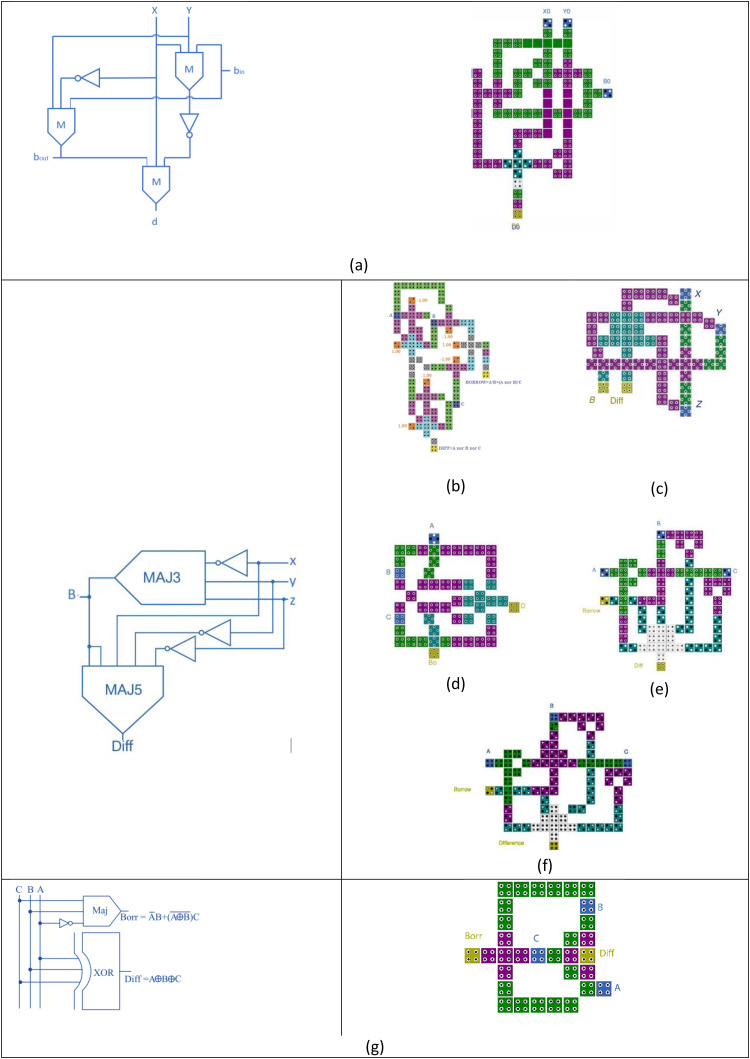
The previous QCA circuits of subtractor given in (a) [11], (b) [12], (c) [13], (d) [14], (e) [15], (f) [16] and (g) [17]).

Moving toward more area-efficient architectures, Labrado and Thapliyal [13] developed a single-layer subtractor layout, requiring only 63 QCA cells and taking 0.05 µm² of area while maintaining a latency of 0.75 clock cycles. Jaiswal and Sasamal [14] followed the same approach and introduced a new structure. Their structures consisted of 53 cells and occupied 0.047 µm², delivering a similar latency of 0.75 clock cycles. Continuing this trend, Raj and Gopalakrishnan [15] presented a single-layer full subtractor based on a 5-input majority gate, requiring 84 cells and occupying an area of about 0.08 µm². In another recent contribution, Vanaraj et al. [16] designed a coplanar subtractor utilizing 87 QCA cells and an area footprint of 0.09 µm². Bahar et al. [17] presented a highly compact architecture, using a combination of a 3-input XOR gate and a 3-input majority gate. This new design used only 32 cells, occupied an area of only 0.0287 µm², and achieved a lower latency of 0.5 clock cycles. The above subtractor circuits are illustrated in Fig 3.

### Dual-function adder/subtractor designs

**Fig 4:**
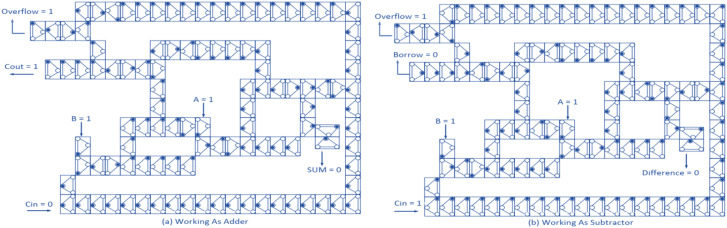
The 3-dot cell based adder and subtractor circuit suggested in [18]).

To overcome the limitations of using separate adder and subtractor units, various researchers have proposed unified architectures capable of handling both operations within a single circuit. Also, when designing complex systems such as arithmetic and logic units (ALUs), the use of separate adder and subtractor modules can lead to increased circuit complexity and propagation delays. To address this, several researchers have proposed compact adder/subtractor architectures, supporting dual-mode operation within a unified architecture. For example, Bardhan et al. [18] presented an adder-subtractor design using 3-dot QCA technology as shown in Fig 4, consisting of 109 cells and occupying 0.79 µm² of area. Barughi and Heikalabad [19] proposed a single-bit architecture based on multilayer wire crossing, which used 90 cells, occupied an area of about 0.6 µm², and exhibited a latency of 3 clock cycles. A more reversible approach was introduced by Ahmad et al. [20], whose single-layer reversible full adder/subtractor circuit required 121 QCA cells, 0.14 µm² of area, and had a latency of 1.25 clock cycles.

A different method was introduced by Mosleh [21], who used the MV32 majority gate to develop a controlled full adder/subtractor. This design required 51 cells, covered an area of 0.05 µm², and had a latency of 1.25 clock cycles. More recently, Raj et al. [22] proposed a dual-function adder/subtractor based on majority logic using 75 cells and occupying 0.09 µm², with a latency of 0.75 clock cycles. Similarly, Marshal et al. [23] presented two versions of a controlled adder/subtractor: the first was a single-layer design with 54 cells and a 0.06 µm² area; the second used multilayer crossover techniques, using only 38 cells and occupying only 0.03 µm², with a latency of 0.5 clock cycles.

Recently, the potential of QCA technology in terms of cell shape and electron repulsion has been exploited to construct logic gates and circuits that operate without relying on Boolean rules. This has been done to reduce the area and number of cells required, thus lowering the cost. Several researchers have followed this advantage, constructing full adders that combine Boolean rules with this unique feature. Al-Tarawneh [24] presented dual compact structures for a full adder-subtractor in both single-layer and multi-layer configurations. The single-layer structure consists of 15 cells and takes an area of about 0.012 µm² with a latency of 0.5 clock cycles.

A new design was recently proposed by Reshi et. al [25]. The block diagram used in this study was slightly different from the diagrams used in previous studies. Although this study is one of the most recent publications on this topic, it did not surpass the design presented by [24] in all metrics.

**Fig 5:**
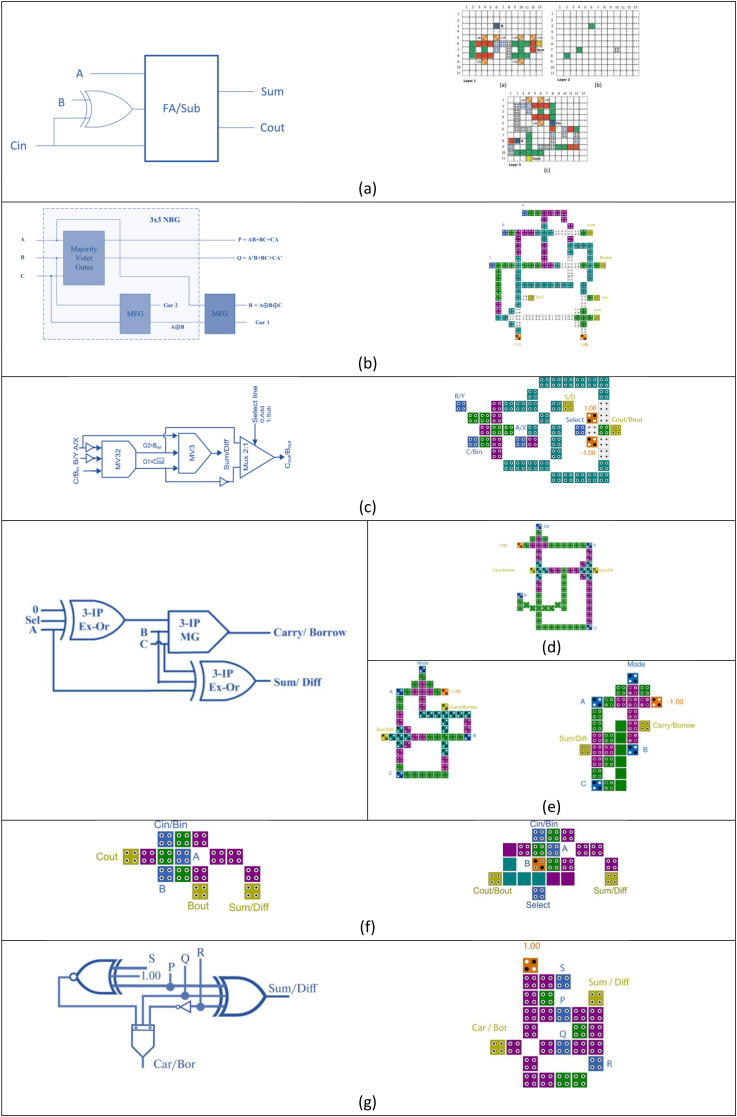
The previous dual-function adder/subtractor circuits given in (a) [19], (b) [20], (c) [21], (d) [22], (e) [23], (f) [24] and (g) [25]).

The above dual-function structures are given in Fig 5. In this work, this compact structure has been modified using another feature of the technique to obtain a new, more compact structure.

These research efforts demonstrate a clear path toward smaller, faster, and more integrated QCA designs. The challenge now is to push these optimizations even further, particularly by balancing functionality, area reduction, and energy efficiency within a single structure.

### Summary and research gap

Despite notable advancements, existing designs still face challenges such as increased complexity, multilayer requirements, or high cell counts, highlighting the need for simpler and more efficient solutions.

In this context, the current work presents a unique design for a QCA adder/subtractor circuit, benefiting from the ability to move the cells by 10 nm instead of 20 nm, which reduces the number of cells required and the occupied area, thus reducing the cost. The proposed design introduces a new cell arrangement to achieve dual functionality within a single-layer design, somewhat similar to that proposed by Altarawneh [24] researchers. By prioritizing both cost and efficiency, this design contributes to a practical step toward developing scalable and manufacturable QCA-based computer circuits.

## Proposed design

The primary objective of this work is to design a compact, energy-efficient QCA circuit that can perform full addition and subtraction operations within a unified structure. Unlike traditional approaches that replicate separate logic units for each operation, the proposed design is based on a single logic framework.

The design is based on majority gate logic, which forms the backbone of most QCA circuits. In QCA, a majority gate with three inputs (A, B, C) produces an output of 1 if at least two of the inputs are 1, expressed as given in Eq 1:


Maj(A,B,C)=AB+AC+BC (1)

Using majority gates, basic logic functions such as AND/OR can be implemented with minimal overhead.

Carry (when addition mode) is explained in Eq 2:


$${\text{C}}_{\text{out}}=\text{AB}+\text{A}{\text{C}}_{\text{in}}+\text{B}{\text{C}}_{\text{in}}=\text{Maj}(\text{A},\text{B},{\text{C}}_{\text{in}})$$ (2)

For this design, we constructed the sum/difference (S/D), carry and borrow outputs using the following equations:

Sum/ Difference shown by Eq 2:


$$\text{S}/\text{D}=\text{A}⊕\text{B}⊕{\text{C}}_{\text{in}}$$ (3)

**Fig 6:**
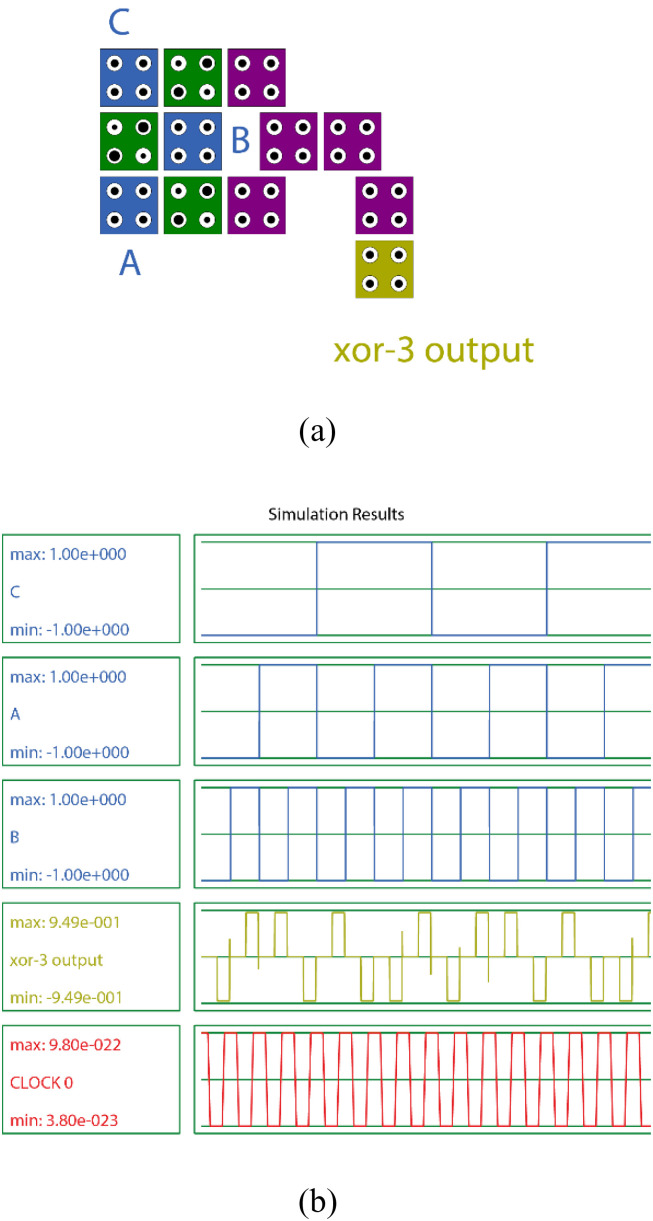
The proposed design of 3-bit XOR gate (a) QCA layout and (b) output waveform.

**Fig 7:**
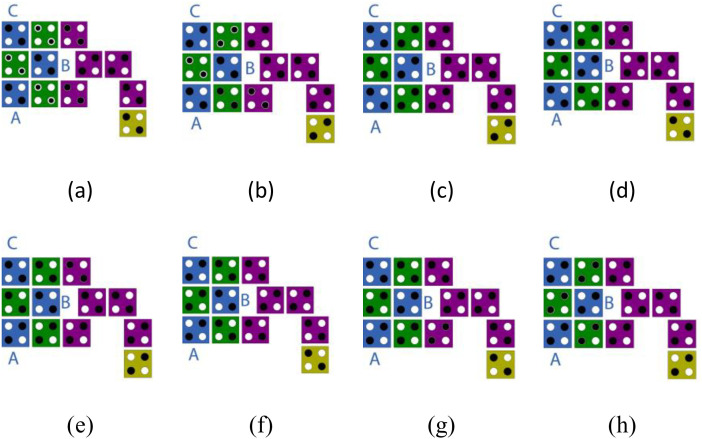
The transition states of cells for the proposed block in order, from state (a) 000 to state (h) 111.

In this research, a new design for a three-bit XOR gate is proposed. This design is characterized by its simplicity, consisting of only 12 cells. Fig 6 illustrates the proposed design, along with the input and output waveforms. This design adheres to the standard truth table for a three-bit XOR circuit. Fig 7 shows all the cell states of the proposed design relative to the standard table of the XOR gate shown in Table 1, thus illustrating its function..

**Table 1:** The truth table of a typical 3-bit XOR gate.

A	B	C	Out = (A ⊕ B ⊕ C)
0	0	0	0
0	0	1	1
0	1	0	1
0	1	1	0
1	0	0	1
1	0	1	0
1	1	0	0
1	1	1	1

The borrowed output cell can be taken from the potential of the QCA, which results from the principle of electron repulsion, where:

Borrow (when subtraction mode) depicted by Eq (4):


$${\text{B}}_{\text{out}}=\overset{―}{\text{A}}\text{B}+\overset{―}{\text{A}}{\text{C}}_{\text{in}}+\text{B}{\text{C}}_{\text{in}}$$ (4)

**Fig 8:**
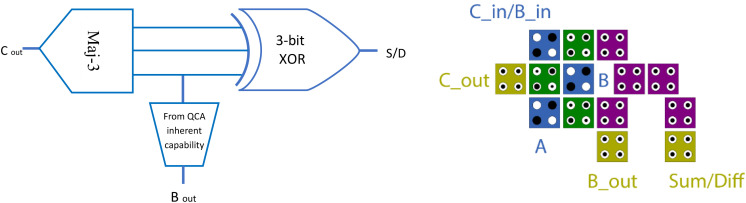
The proposed block diagram and QCA configuration of dual function full adder-subtractor.

The proposed full adder integrates XOR, majority, and inverter components into a single-layer configuration to achieve minimal area, cell count, and circuit cost without compromising circuit efficiency. The entire circuit is implemented using QCADesigner 2.0.3, with a cell size of 18 nm × 18 nm and intercell spacing of 2 nm. The proposed block diagram and QCA layout are shown in Fig 8.

The proposed configuration consists of only 14 cells, a low latency (0.5 clock cycles), zero crossover, and an occupied area of approximately 0.009 µm². This design not only simplifies the integration of arithmetic functions but also reduces the design complexity of larger QCA-based systems, such as arithmetic and logic units or signal processors.

## Simulation result and comparison

**Fig 9:**
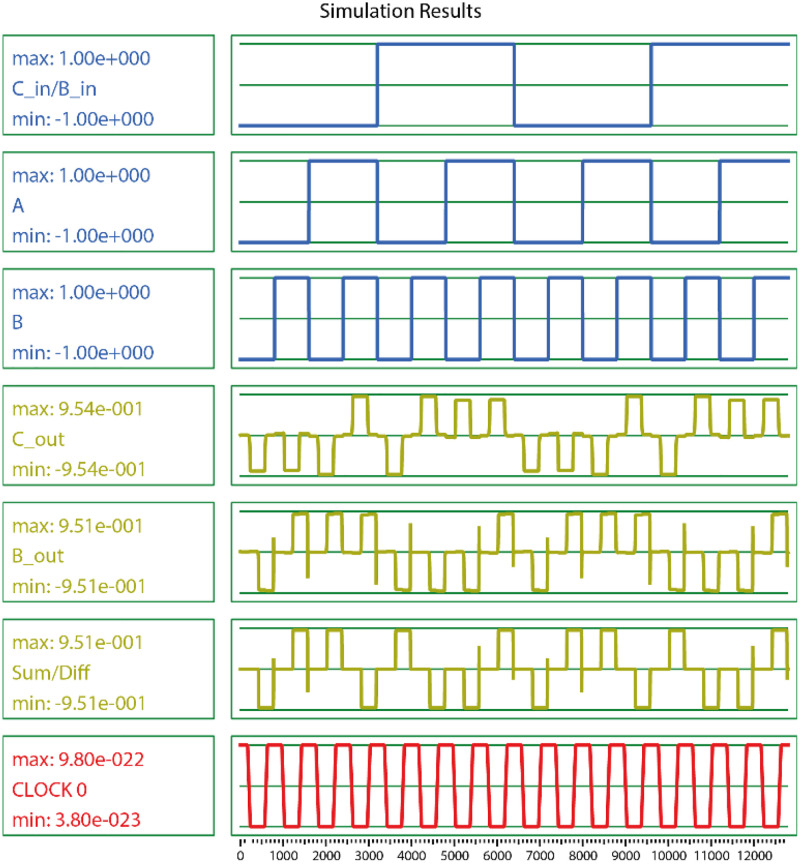
Simulation output for the proposed unit.

To validate the functionality and efficiency of the proposed full adder/subtractor circuit, comprehensive simulations were performed using QCADesigner version 2.0.3, a widely accepted tool for QCA layout design and verification. The default values of the simulation software were used to simulate the proposed circuit. The proposed layout was tested using both the coherence and bistable simulation engines in QCADesigner without any errors, as shown in Fig 9. A waveform analysis confirmed the stability of the signal transitions across clock zones, and no metastable states were detected during switching. The proposed structure functioned reliably under both modes.

Cost calculation in QCA is of paramount importance, as the cost function has been presented in different approaches. The most equitable approach among those presented previously is the one presented in [26], which includes the layers required for implementation, as well as area, number of cells, and delay time as given in Eq 5.


Circuit~cost=CLF*cell~count*Area*Delay (5)

where CLF is the cells and layout factor:

CLF = 1: for single-layer/ normal cells;

= 2: for single-layer/normal and rotated cells;

= 3: for multi-layer/ normal cells; and

= 4: for multi-layer/normal and rotated cells

The proposed architecture demonstrates significant improvements, offering 7%, 25%, and 30% reductions in cell count, area, and cost, respectively, compared to the best-reported design. Table 2 summarizes the comparative metrics with selected designs from previous studies.

**Table 2:** Comparison with existing QCA Adder/Subtractor designs.

Reference	Dual-mode supported	Cell Count	Area (µm²)	Delay (Latency)	Crossover Type	Circuit Cost
(6)	No	192	0.20	Not applicable	Rotated cells	307.2
(7)	No	145	0.17	1 clock cycle	Rotated cells	197.2
(9)	No	73	0.04	0.75 clock cycles	Multilayer	26.28
(10)	No	41	0.04	0.5 clock cycles	Rotated cells	6.56
(19)	Yes	90	0.6	3 clock cycles	Multilayer	1944
(20)	Yes	121	0.14	1.25 clock cycles	Logical crossover	84.7
(21)	Yes	51	0.05	1.25 clock cycles	None	12.75
(22)	Yes	75	0.09	0.75 clock cycles	Rotated cells	40.5
(23)	Yes	38	0.03	0.5 clock cycles	Multilayer	6.84
(25)	Yes	27	0.018	0.5 clock cycles	None	0.927
(24)	Yes	15	0.012	0.5 clock cycles	None	0.36
Proposed	Yes	14	0.009	0.5 clock cycles	None	0.252

Furthermore, although QCADesigner 2.0.3 does not directly calculate power dissipation, the reduced cell count and reduced circuit area indirectly translate to lower dynamic power requirements. Therefore, an additional comparison will be conducted using QCAPro to quantify energy metrics for further verification.

### Energy dissipation analysis

In QCA circuits, energy dissipation remains a central performance concern, even as the technology shows remarkable promise for low-energy computing. Unlike CMOS technology, which is dominated by continuous current flow, QCA relies on electron polarization within quantum dots, reducing the energy required for switching events. Yet this efficiency is tempered by intrinsic leakage mechanisms. Quantum tunneling is a primary source, as electrons may unintentionally tunnel between neighboring dots outside the desired polarization, leading to unwanted dissipation. Thermal fluctuations add another layer of complexity, occasionally destabilizing cell states and pushing the system to expend extra energy to recover stability. Clocking imperfections can also introduce incomplete or delayed switching, compounding energy losses over time. These subtle but unavoidable effects remind us that, despite QCA’s elegance and efficiency, it still faces practical hurdles. Acknowledging these leakage pathways offers a more complete picture of circuit behavior and points the way toward refinements that can unlock QCA’s full potential. To thoroughly evaluate the energy performance of our proposed full adder/subtractor design, we utilized QCAPro, a specialized simulation tool widely recognized for its ability to estimate power dissipation in QCA circuits accurately. Using this tool, we performed comprehensive simulations under standard operating conditions given below, examining the switching power and leakage power across all cells and clock zones.

Temperature: 2 K

Tunneling energies: 0.5 Ek, 1 Ek, 1.5 Ek

Clocking zones: Four-phase clocking with standard delay

Input patterns: All binary combinations of the 3 input bits (A, B, Cin) were simulated using a truth-table-driven input vector.

The results showed that our proposed design not only achieves functional accuracy and reduced area, but also significantly outperforms all previously published designs in terms of total energy dissipation.

Compared to prominent recent works, our circuit consumed the least amount of energy. This energy-efficient behavior is primarily attributed to the minimal number of active cells, optimized wire lengths, and clock zone management, all of which help reduce unnecessary transitions and polarization losses. These results demonstrate the practicality of our design for future QCA-based systems, particularly in environments where thermal constraints and low-power operation are critical. By focusing not only on structural and logical efficiency but also on energy conservation, this design represents a significant advance toward scalable, low-power quantum dot-based cellular automation architectures.

To accurately evaluate its energy profile, we performed simulations using the QCAPro software across three distinct tunneling energy levels 0.5 Ek, 1 Ek, and 1.5 Ek, under a controlled cryogenic temperature of 2 K. This evaluation allowed for a detailed comparison of the proposed design with other established QCA collectors in terms of leakage and switching power. As illustrated in Table 3, the switching energy consumed during state transitions remains consistently low across all three tunneling energy levels. Furthermore, when comparing total dissipated energy, our proposed design outperforms all previously reported architectures that have undergone similar power dissipation evaluations.

**Table 3:** A comparative analysis on energy consumption of QCA adder/subtractor.

Full Adder/Subtractor	Average of leakage energy dissipation (mev)			Average of switching energy dissipation (mev)			Total energy consumption (mev)		
	0.5E_k_	1E_k_	1.5E_k_	0.5E_k_	1E_k_	1.5E_k_	0.5E_k_	1E_k_	1.5E_k_
(20)	40	117	206	152	131	111	192	248	317
(21)	21	54	91	42	35	28	63	89	119
(22)	38	109	189	111	94	78	149	203	267
(23)	21	59	102	67	56	46	88	115	148
(24)	6	14	23	12	9	8	18	23	31
(25)	11	27	45	20	16	13	31	43	58
Proposed	5	12	21	12	10	8	17	22	29

**Fig 10:**
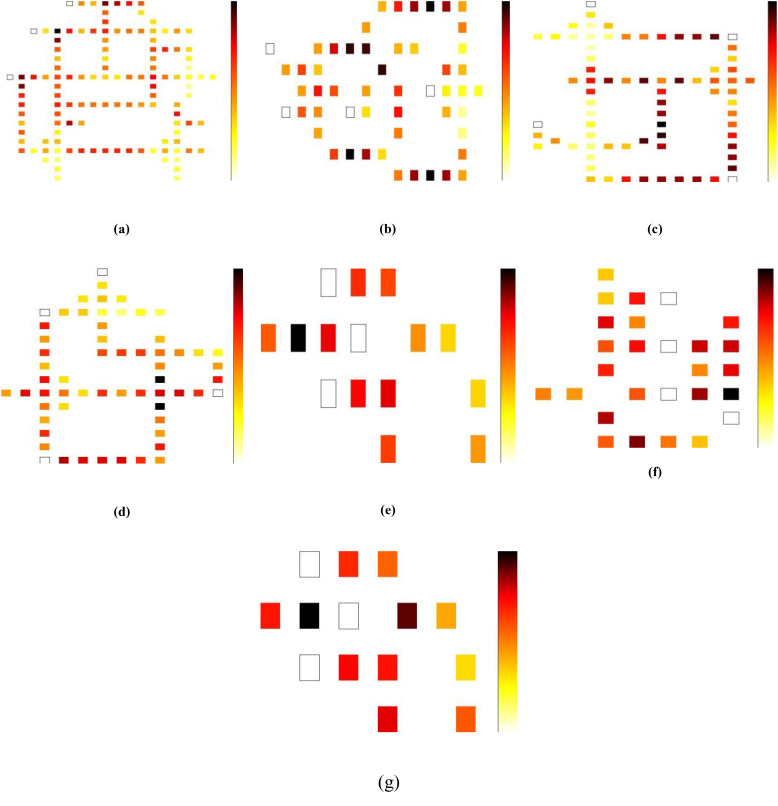
Energy dissipation maps at 2K temperature and tunneling energy of 0.5 Ek for QCA full adder/subtractor given in (a) [20], (b) [21], (c) [22], (d) [23], (e) [24], (f) [25] and (g) proposed.

While the total energy consumption increases with increasing tunnel power, as expected, the proposed design exhibits a more gradual increase in energy consumption compared to previous designs, indicating improved energy efficiency. This robustness can be attributed to its optimized geometric layout and minimal inter-cell transitions. Quantitatively, the total power savings achieved by the proposed design reach approximately 6%, 4%, and 6% at tunneling energies of 0.5 Ek, 1 Ek, and 1.5 Ek, respectively, compared to their counterparts (The latest same circuits that include energy calculations). This reduction not only confirms the energy-aware nature of the architecture but also enhances its suitability for low-power and thermally constrained applications in future nanocircuits. The energy dissipation maps at 2 K temperature and 0.5 Ek tunneling energy for the previous QCA adder/subtractor with the proposed structure are shown in Fig 10.

## Conclusion

This paper presented a novel Quantum-dot Cellular Automata (QCA) design that combines full adder and subtractor functionalities within a single, compact circuit architecture. By integrating a control input to toggle between arithmetic modes, the proposed design eliminates the need for redundant hardware, offering an efficient solution for low-power and high-density digital computation at the nanoscale.

Through detailed simulation using QCADesigner, the circuit demonstrated correct logical behavior for all input combinations and showed improved metrics over existing designs, including reduced cell count, smaller area, and lower latency. Additionally, the use of a crossover-free, coplanar layout enhances the physical feasibility of the design in future fabrication processes.

The proposed structure is well-suited for integration into larger QCA-based processing units such as arithmetic logic units (ALUs) and signal processing cores. As QCA technology continues to evolve, such multifunctional and optimized logic elements will be essential in building scalable, energy-aware computing architectures beyond the limits of traditional CMOS.

The novelty of the proposed architecture lies in achieving a simple, power-efficient, and single-layer cell design, setting a new standard in QCA circuit design.

In future work, we aim to extend the proposed design into multi-bit architectures and evaluate its performance under realistic physical conditions using tools such as QCADesigner-E for energy profiling and QCAPro for fault analysis.
